# Potential habitat suitability of *Candidatus* Liberibacter asiaticus and genetic diversity of its prophages across China

**DOI:** 10.1128/spectrum.00633-24

**Published:** 2024-09-24

**Authors:** Ping You, Jun Zhou, Amir Muhammad Bilal, Minli Bao, Jin Yang, Shujie Fang, Xiang Li, Long Yi

**Affiliations:** 1 School of Life Science, Gannan Normal University, Ganzhou, Jiangxi, China; 2 National Navel Orange Engineering Research Center, Ganzhou, Jiangxi, China; 3 South China Botanical Garden, Chinese Academy of Sciences, Guangzhou, Guangdong, China; Universidad Nacional Autonoma de Mexico - Campus Morelos, Cuernavaca, Mexico

**Keywords:** Huanglongbing, *Candidatus *Liberibacter asiaticus, diversity, MaxEnt, prophage

## Abstract

Huanglongbing (HLB) is a severe citrus disease in China caused by *Candidatus* Liberibacter asiaticus (*C*Las). Since its initial identification, the pathogen has spread to 10 mainland provinces in China and caused devastating loss. Three distinct prophage types have been identified in *C*Las; however, their distribution and diversity in China remain inadequately understood. In this study, we collected 500 *C*Las samples from 10 provinces in China, employing three specific genomic loci to identify prophage types. Subsequently, Sanger sequencing was employed to analyze the genetic diversity of prophage within populations of *C*Las in China. In addition, the MaxEnt model optimized by the ENMeval software package, was used to predict the habitat suitability of populations of *C*Las and assess the potential impact of future climate change on its distribution in China. Our analysis revealed that type 2 prophage is the most prevalent, accounting for 55% in China. Among the 10 provinces tested, *C*Las populations in Yunnan and Sichuan demonstrated higher genetic diversity. Further analysis reveals that *C*Las populations harboring type 1 prophage remain relatively stable, whereas those carrying type 2 and type 3 prophages undergo population expansion. Furthermore, our predictive models indicate that the presently suitable habitat for *C*Las populations is concentrated in the southern and certain central regions of China, with an anticipated expansion under future climate change conditions. Presently, the center of populations of *C*Las characterized by favorable living conditions is situated in Zunyi City, Guizhou Province. Nevertheless, a projected trend indicates a shift toward the northeast, particularly targeting Tongren City in the foreseeable future.

**IMPORTANCE:**

This study offers significant insights into the distribution and genetic diversity of three types of prophages associated with *Candidatus* Liberibacter asiaticus (*C*Las) in China. Our predictions underscore the implications of climate change on the future distribution of *C*Las. These findings contribute to a better understanding of Huanglongbing management strategies and can facilitate the development of effective measures to control the spread of this devastating disease within the citrus industry.

## INTRODUCTION

Citrus is the most widely planted and highest-yielding fruit globally. China stands out as a major producer of citrus fruits, with cultivation spanning across 19 provinces (1). However, the citrus industry is facing a significant global threat from Huanglongbing (HLB), a disease caused by *Candidatus* Liberibacter asiaticus (*C*Las), affecting citrus cultivation worldwide, including that in China (2). *C*Las is a Gram-negative pathogen first discovered in Guangdong Province in the 1920s, and it has been spreading northward ever since (3–5). HLB has been documented in 349 counties across 10 provinces in mainland China. Infected citrus trees manifest symptoms, such as leaf yellowing, deformed fruits, reduced yield, and, in severe cases, eventual mortality (6–9). Presently, there are no effective management methods or resistant varieties available to combat this disease. Therefore, the most effective prevention and control measures against the disease involve the removal of infected trees and the control of vector spread (10–12).

Prophages are viruses that parasitize and replicate within the bacterial hosts, playing a crucial role in maintaining the balance of microbial communities and ecosystem functions. Numerous studies have identified three types of prophages associated with *C*Las: SC1 (classified as type 1), SC2 (classified as type 2), and P-JXGC (classified as type 3) (13, 14).

Type 1 prophages are involved in the formation of phage particle lysis cycles, whereas type 2 prophages participate in the pathogenic mechanism of *C*Las through lysogenic conversion. Type 3 prophages carry a restriction–modification system to defend against foreign phage invasion. The functional annotation of genes reveals that type 1 and 2 prophages contain genes encoding multiple virulence factors, potentially enhancing *C*Las pathogenicity. Grafting experiments were conducted on periwinkle hosts and various citrus varieties using *C*Las strains carrying types 1 and 2 (15, 16). These studies have revealed a distinct pathogenicity induced by the two types of prophages in *C*Las strains, with type 1 demonstrating a higher degree of pathogenicity.

*Diaphorina citri* (Asian citrus psyllid) is the primary vector of *C*Las (17). Upon feeding on leaves infected with *C*Las, *D. citri* becomes infected and subsequently spreads the pathogen throughout its entire lifecycle (18). Citrus sprouts exhibit vigorous growth during summer and autumn, creating favorable conditions for the proliferation and spread of *C*Las and *D. citri* (19, 20). Several studies have demonstrated that the incidence rate of *C*Las is correlated not only with the season but also with the altitude (21–24). In recent years, factors, such as climate warming and the transportation of seedlings, have contributed to the gradual northward spread of *D. citri* and *C*Las. *C*Las has now extended to Yibin City in Sichuan Province, posing an urgent threat to the citrus industry in Sichuan Province and Chongqing City (25).

The geographic environment significantly influences the genetic diversity of *C*Las. Previous studies have shown that the diversity of Chinese *C*Las is correlated with the geographical distribution, and distinct genetic variations exist among *C*Las populations across different regions of China (16, 26). As *C*Las continues to spread within the country, it is crucial to comprehend its population structure in various geographic regions (27, 28). This understanding will aid in the epidemiological investigations of HLB in citrus and form a basis for control strategies. Genomic sequence analysis reveals that the *C*Las prophage exhibits a greater nucleotide-level variation compared to the chromosome, making it ideal for studying the *C*Las genetic diversity (16, 27, 29). Ecological niche modeling (ENM) has emerged as a valuable tool in recent years, integrating analysis with population genetics. Before making spatial or temporal predictions, ENM models climate envelopes based on species occurrence records and climate data to forecast the impact of future climate scenarios (30–32).

Ecological niche modeling can predict future habitat changes, while genomic sequence data can identify adaptive genetic diversity. In summary, these methods provide insights into local adaptation profiles, offering valuable guidance for species management strategies (33).

The primary objective of this study is to explore the genetic variation patterns within the Chinese *C*Las population and assess habitat suitability. These findings collectively aim to inform control measures for *C*Las in China. We conducted extensive surveys and sampling across 10 provinces, collecting a total of 500 *C*Las samples. The analysis involved three specific prophage genes, SC1-gp035, SC2-gp030, and P-JXGC-gp08, and aimed at investigating the population evolution, genetic diversity, and expansion history (16). Furthermore, we employed the maximum entropy model (MaxEnt) (34) to analyze the key factors influencing the geographical suitability pattern of *C*Las. We predicted the current habitat suitability of *C*Las and its potential geographic distribution under different climate change scenarios. Specifically, we simulated the response of *C*Las to global warming between the 2050s and 2070s under four shared socioeconomic pathway (SSP) conditions (35). The research findings will provide a robust theoretical foundation for developing effective prevention and control strategies for *C*Las.

## RESULTS

### Distribution of different prophages of *C*Las

**Fig 1:**
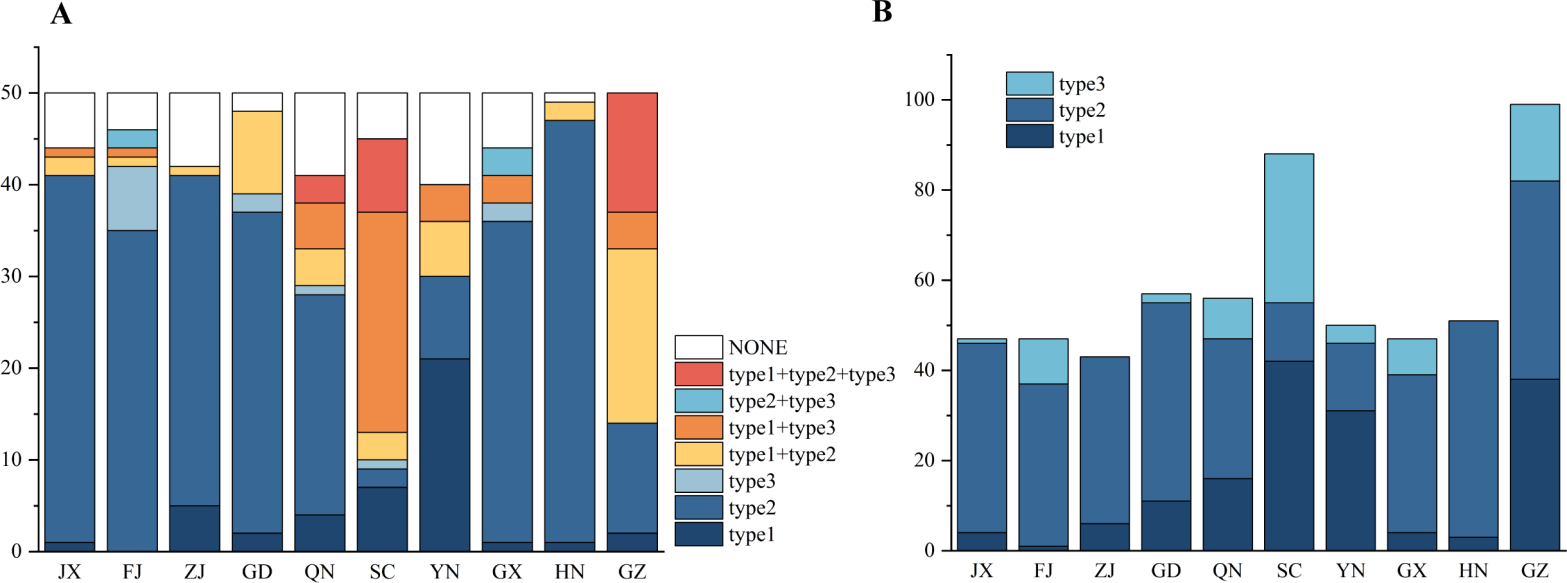
Distribution of various combinations of prophages in the *C*Las samples collected in China. (**A**) Proportional distribution of different combinations of prophages within distinct populations. (**B**) Proportional distribution of the three types of prophages across different populations.

Using specific PCR markers targeting three known prophage types, we successfully identified eight types of prophage combinations (Fig. 1; Table S1). Within the sampled population, 275 *C*Las samples were found to exclusively harbor type 1 prophage, constituting 55% of the entire cohort. Moreover, 44 samples exclusively contained type 2 prophage, accounting for 8.8%, and 13 samples carried type 3 prophage exclusively, representing 2.6% of the total.

Furthermore, we observed the coexistence patterns of prophages in the *C*Las strain. Specifically, 46 samples showed the presence of both the type 1 and type 2 prophages, while 42 samples exhibited co-infection with the type 1 and type 3 prophages. Additionally, five samples demonstrated a co-occurrence of the type 2 and type 3 prophages, and 24 samples displayed simultaneous infection with all three types of prophages. Notably, 51 samples did not exhibit detectable prophages. The frequencies of different prophages varied across provinces, with Yunnan, Sichuan, and Guizhou predominantly associated with the type 1, type 1 + type 3, and type 1 + type 2 prophages, respectively, each exceeding 38%. In contrast, the Hainan, Guangxi, Guangdong, Fujian, Zhejiang, Hunan, and Jiangxi provinces showed a predominant presence of the type 2 prophage, each exceeding 48%.

### Genetic diversity of three prophage types of *C*Las

The gene sequences of SC1-gp35, SC2-gp30, and P-JXGC-gp08 were successfully obtained, with lengths of 903 bp, 736 bp, and 816 bp, respectively. The analysis of the nucleotide sites in SC1-gp35 revealed 865 conserved sites and 38 variable sites, resulting in 15 haplotypes (Table 1). The *C*Las populations of the Sichuan and Guangdong provinces exhibited the highest number of haplotypes at seven each, with Guangdong showing the highest haplotype diversity of 0.89091, followed by Sichuan with 0.41812. Regarding the nucleotide sites of SC2-gp30, 730 sites were conserved with only six variable sites, resulting in seven haplotypes. Intriguingly, variation was detected in the samples from Yunnan, Guizhou, and Sichuan, while no variation was found in the samples from the other seven provinces. Sichuan and Yunnan exhibited relatively high haplotype diversities of 0.53846 and 0.36190, respectively, while Guizhou had a lower haplotype diversity of 0.04545. Concerning the nucleotide sites of P-JXGC-gp08, 811 sites were conserved with five variable sites, resulting in six haplotypes. Guangxi, Hainan, and Jiangxi each exhibited three haplotypes, while Sichuan exhibited two haplotypes. No variable sites were detected in the other six provinces (Table S2).

**TABLE 1:** Genetic diversity of various prophage loci of *C*Las in China

Prophage type	Locus	N	S	H	Hd	Pi	K
Type 1	SC1-gp035	157	38	15	0.24955	0.0014	1.267
Type 2	SC2-gp030	350	6	7	0.04521	0.00006	0.046
Type 3	P-JXGC-gp08	84	5	6	0.138	0.00025	0.213

^*a*^ S: number of polymorphic sites; Hd: haplotype diversity; Pi: nucleotide diversity; H: number of haplotypes; K: average number of pairwise nucleotide differences.

### Phylogenetic tree and haploid network

**Fig 2:**
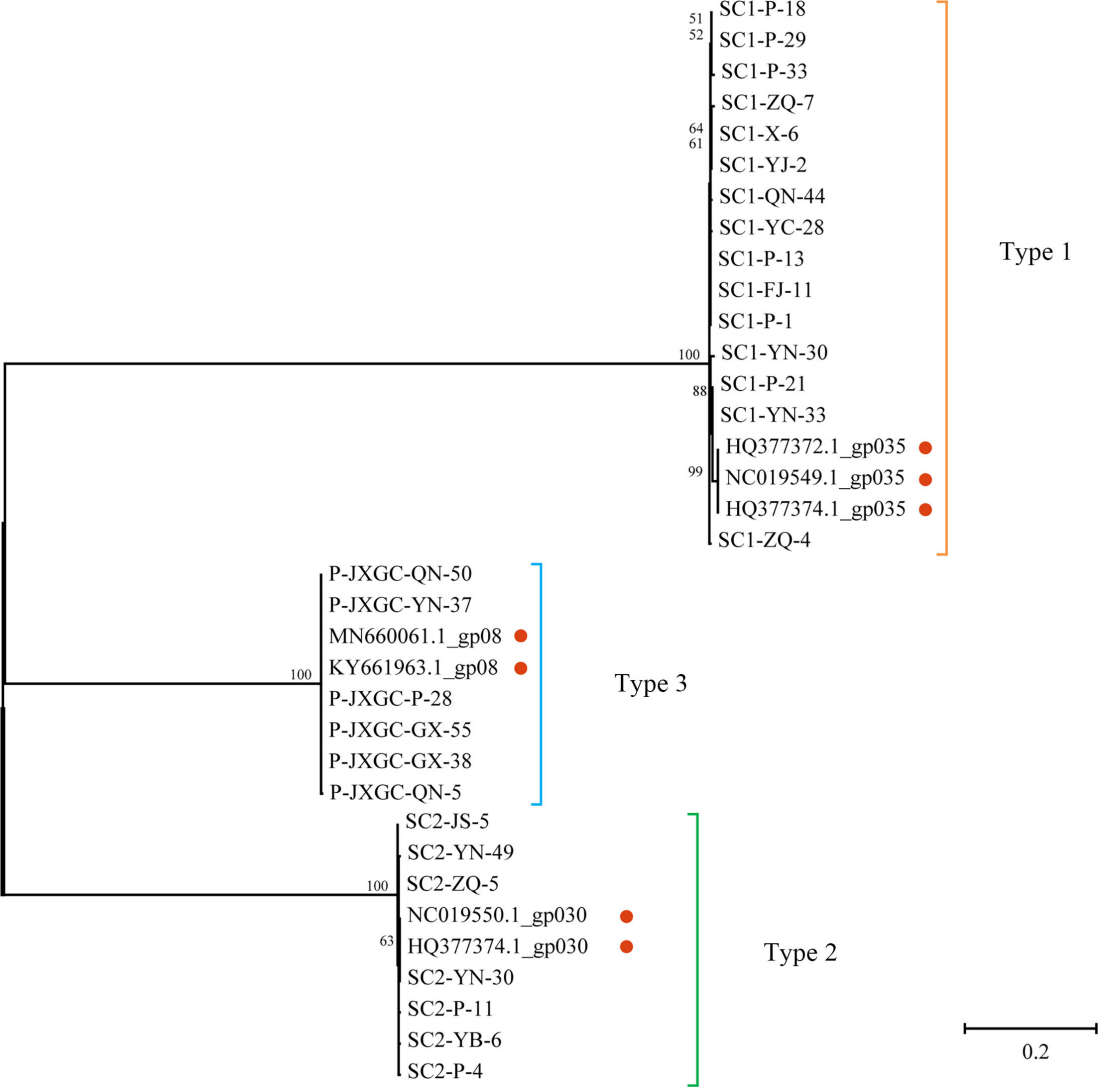
Phylogenetic tree constructed using the representative haplotype sequences of the three types of prophages. Sequences obtained from publicly available phages are highlighted with a red circle.

We have submitted our representative haplotype sequences to the NCBI GenBank database under the accession numbers PP116515–PP116536 and PP116544–PP116549. Specifically, our submission included 15 SC1-035 sequences, seven SC2-gp30 sequences, and six P-JXGC-gp08 sequences. Additionally, we retrieved seven related sequences from the same database: SC1-gp035 (accession numbers: HQ377372.1, NC019549.1, HQ377374.1), SC2-gp030 (accession numbers: NC019550.1, HQ377374.1), and P-JXGC-gp08 (accession numbers: MN660061.1, KY661963.1). Using these sequences, we constructed a phylogenetic tree employing maximum likelihood methods to elucidate the evolutionary relationships among all 35 strains. The resulting tree revealed three distinct clusters with high bootstrap support values, as depicted in Fig. 2.

**Fig 3:**
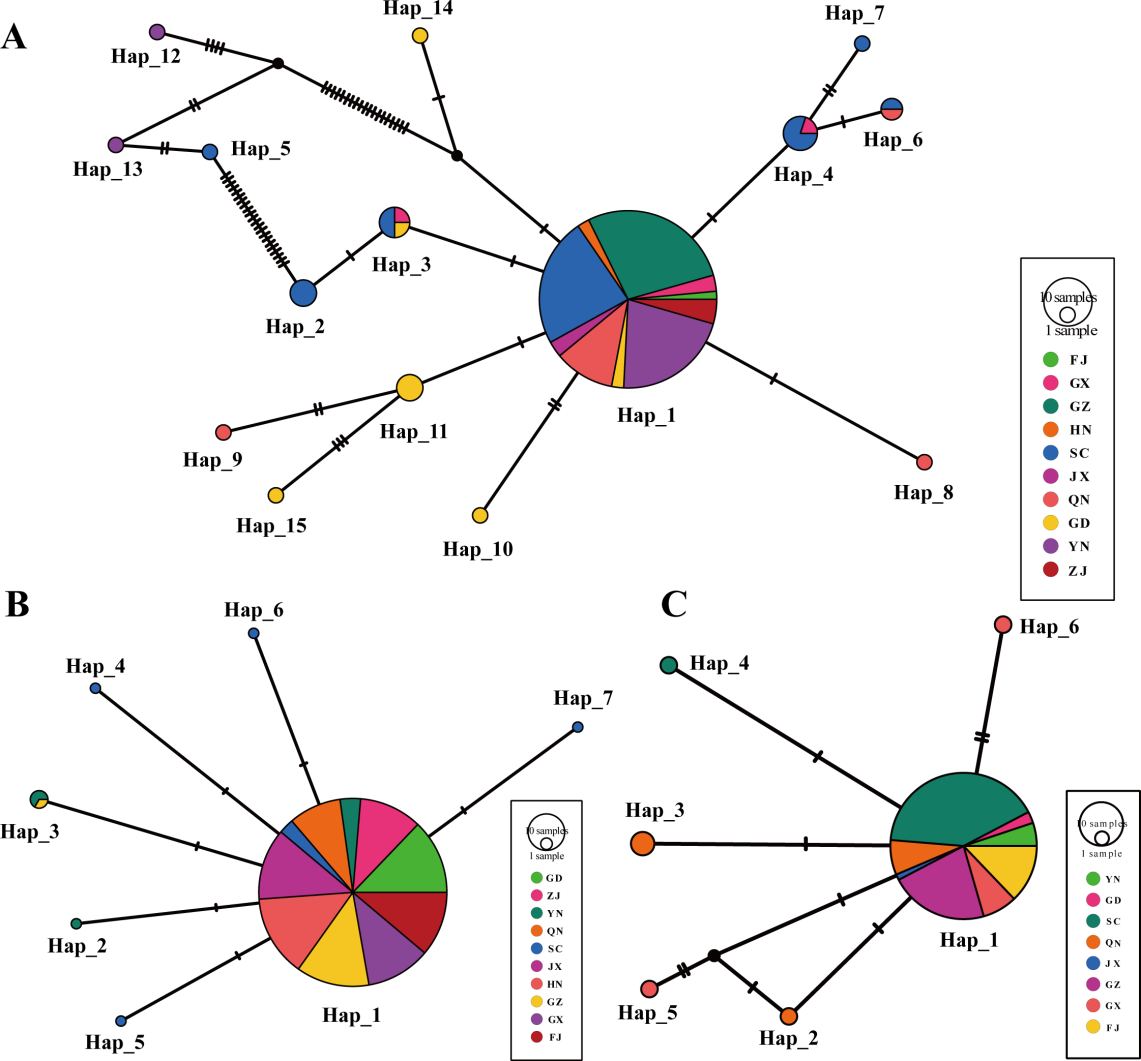
Haplotype networks based on various prophage loci in China: (**A**) SC1-gp035, (**B**) SC2-gp030, and (**C**) P-JXGC-08.

Figure 3 illustrates the evolutionary relationships among different haplotypes, revealing a star-like pattern for SC2-gp030 and P-JXGC-gp08. The central position is occupied by the ancestral haplotype, H1. Notably, there is no discernible clustering of private haplotypes within any population. In contrast, the median-joining network represented by SC1-gp035 shows a notable prevalence of unique mutations. Particularly, the distinct haplotypes Hap_12 and Hap_13 from Yunnan, along with Hap_15 from Sichuan, demonstrate a clustered grouping. This suggests the possibility of a distinct lineage that has significantly diverged from the ancestral haplotype.

### Genetic differentiation and population expansion history inference

The genetic differentiation index (F*st*) is a metric utilized in population genetics to quantify the extent of genetic differentiation among populations. Our results revealed that only SC2-gp030 and P-JXGC-gp08 exhibited F*st* values below 0.05, indicating the lack of significant genetic differentiation between populations. Conversely, the F*st* value for SC1-gp035 was 0.06428, surpassing 0.05 and suggesting a moderate genetic differentiation (Table 2).

**TABLE 2:** Genetic differentiation index and neutrality test of various prophage genes in China

Prophage type	Locus	F*st*	Neutrality test		
			Tajima’s *D*	Fu and Li’s *D*	Fu and Li’s *F*
Type 1	SC1-gp035	0.06428	−2.44812^*b*^	−2.06663^*a*^	−2.67785
Type 2	SC2-gp030	0.01671	−1.80236^*a*^	−4.43935^*a*^	−4.21454^*a*^
Type 3	P-JXGC-gp08	0.02582	−2.16007^*a*^	−4.22408^*a*^	−4.17061^*a*^

^*a*^ *P* < 0.05.

^*b*^ *P* < 0.01.

Neutral analyses and mismatch distributions were employed to infer the demographic history. We calculated the values of Fu and Li’s *D*, Fu and Li’s *F*, and Tajima’s *D* for all three loci, observing significant negative values for SC2-gp030 and P-JXGC-gp08. A mismatch distribution analysis revealed an unimodal distribution for these two prophage types, indicative of a population expansion.

**Fig 4:**
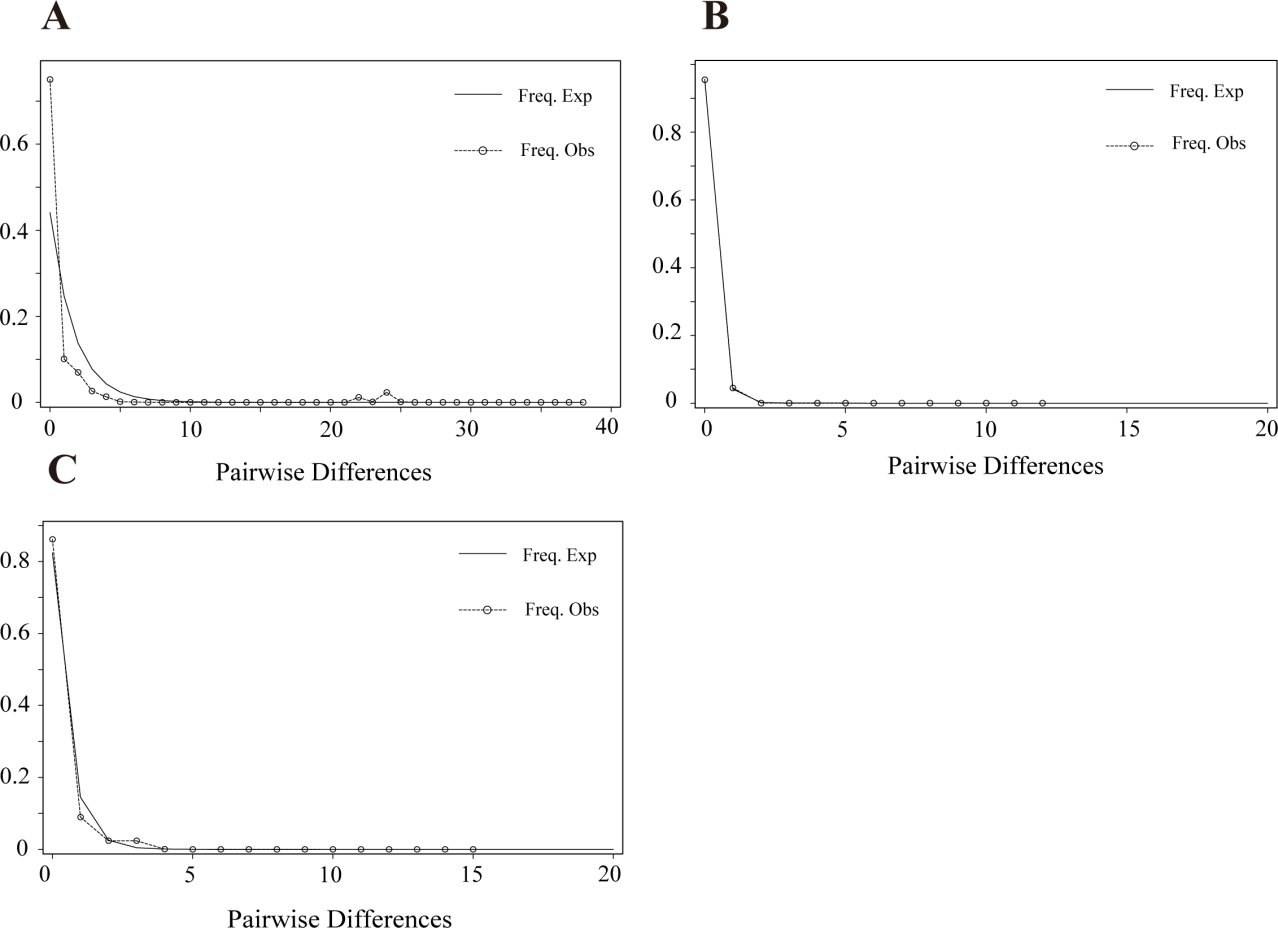
Mismatch distribution curves are based on various prophage loci in China: (**A**) SC1-gp035, (**B**) SC2-gp030, and (**C**) P-JXGC-08.

However, it is crucial to note that SC1-gp035 exhibited significantly negative Tajima’s *D* and Fu and Li’s *D* values, along with a non-significant Fu and Li’s *F* value and a multimodal mismatch distribution curve (Fig. 4). This suggests greater population stability for the type 1 prophage.

### Current habitat suitability zoning of *C*Las

**Fig 5:**
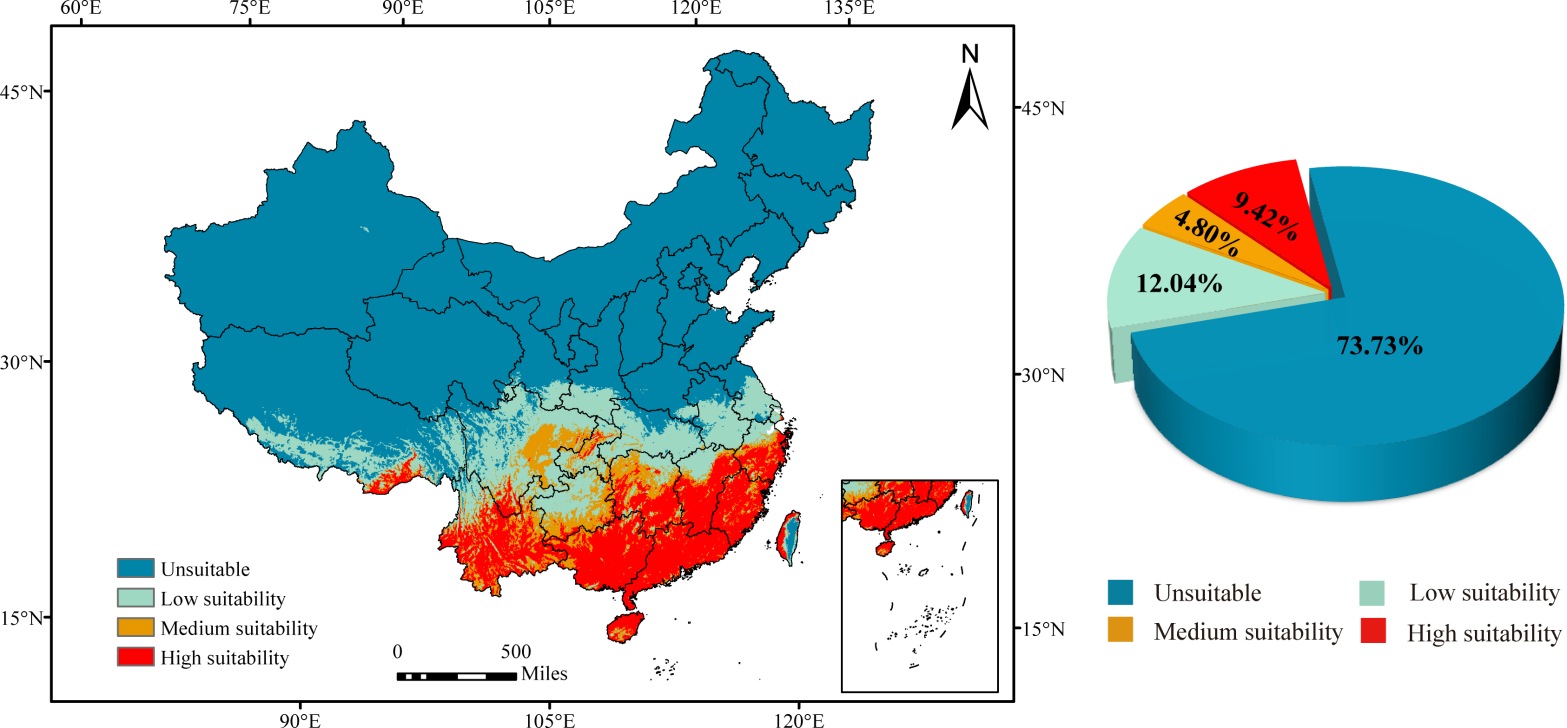
Classification of habitat suitability for *C*Las under the current climate.

The seven environmental variables in the model, particularly bio4 (temperature seasonality) and bio20 (elevation) emerge as the primary influencing factors contributing significantly to the model’s performance with contribution rates of 45.7% and 40.4%, respectively (Table S3). Based on the current climatic conditions, the suitable area for *C*Las covers 26.27% of China’s total land area, while the unsuitable area accounts for 73.73%. The suitable area can be further categorized into three levels: low suitability, medium suitability, and high suitability. The high-suitability region is predominantly found in southern China, encompassing approximately 12.04% of China’s land area. The medium-suitability region is mainly concentrated in provinces, such as Sichuan, Guizhou, Jiangxi, Yunnan, and Xizang, accounting for around 4.80% of China’s land area. Conversely, the low-suitability region is concentrated in central China, particularly in areas like Hubei, Jiangsu, Hubei, and Shaanxi, covering approximately 9.42% of China’s total land area. The percentages are rounded to two decimal places (Fig. 5).

### Potential distributions of *C*Las under different future climate scenarios

**Fig 6:**
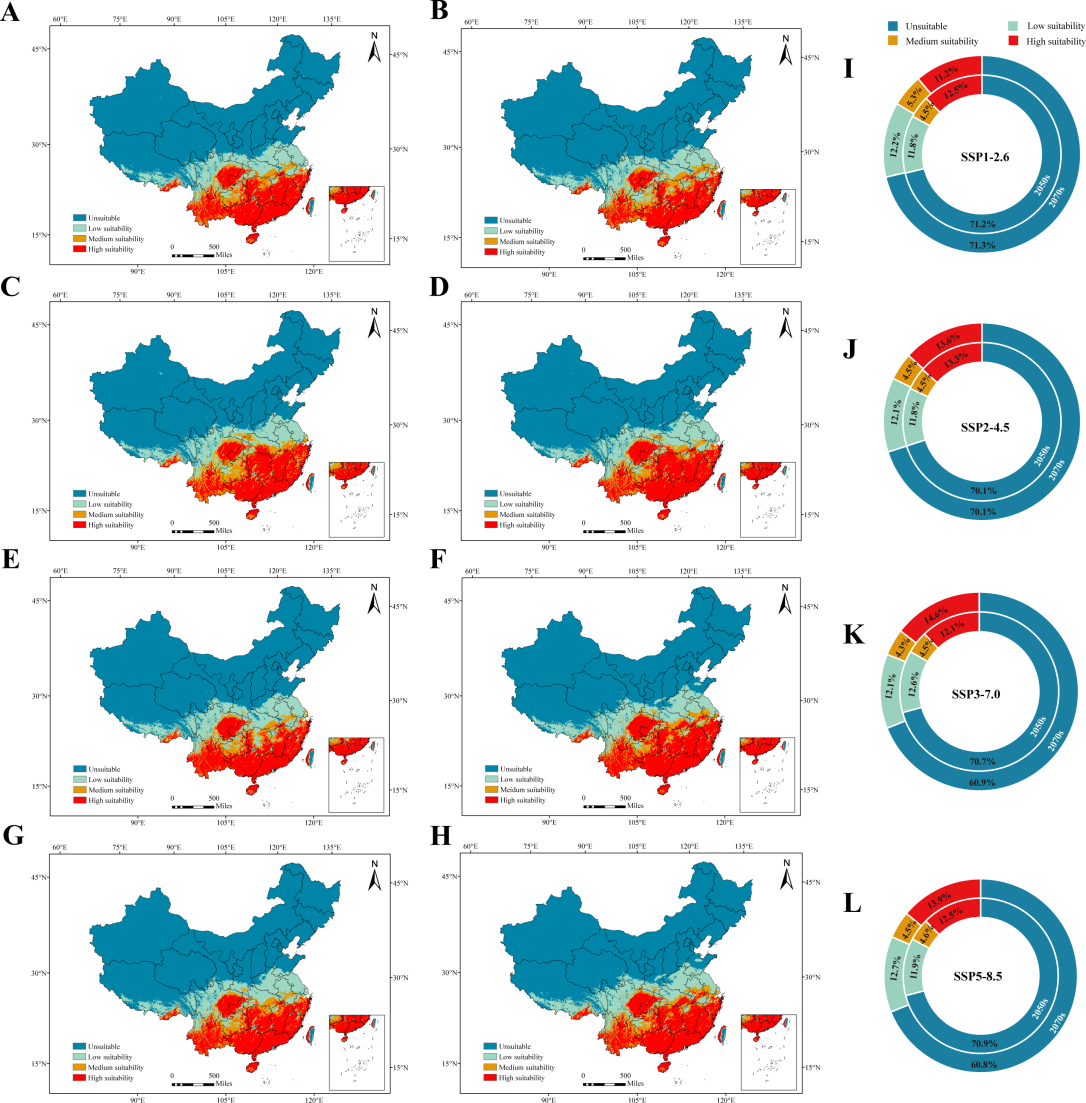
Classification of habitat suitability for *C*Las under current climate scenarios. Panels A–D represent potential habitat suitability in the SSP1-2.6, SSP2-4.5, SSP3-7.0, and SSP5-8.5 scenarios for the 2050s, respectively. Panels E–H represent the potential habitat suitability in the same scenarios for the 2070s. Panels I–L show the percentage of potential suitable and unsuitable areas for *C*Las under the same scenarios for both the 2050s and 2070s in China.

Compared to the current potential distribution areas, the primary suitable regions for *C*Las under future climate conditions will continue to be concentrated in southern China. However, across various future climate scenarios, the suitable growth zone of *C*Las is projected to expand, particularly in highly suitable growth areas. These projections align with the results of calculations based on four concentration regulation pathways for the 2050s and 2070s, as depicted in Fig. 6.

Under the SSP1-2.6 scenario, it is anticipated that the suitable habitat area for *C*Las will increase by 2.56% and 2.44% by the 2050s and 2070s, respectively, compared to the current situation. In the SSP2-4.5 scenario, a similar trend is expected, with the suitable habitat area projected to increase by 3.66% and 3.68% by the 2050s and 2070s, respectively. In the subsp. 3.70 scenario, the anticipated expansion of the suitable habitat area is projected to be 2.99% by the 2050s and 4.75% by the 2070s. Finally, under the SSP5-8.5 scenario, the anticipated increases by the 2050s and 2070s are 2.84% and 4.81%, respectively, compared to the current situation. It is noteworthy that the changes in the *C*Las suitable habitat are relatively modest in the SSP1-2.6 and SSP2-4.5 scenarios for the 2050s and 2070s. However, a more pronounced disparity is observed between the 2050s and 2070s in the SSP3-7.0 and SSP5-8.5 scenarios.

### Path of the centroid of the potential distribution of *C*Las

**Fig 7:**
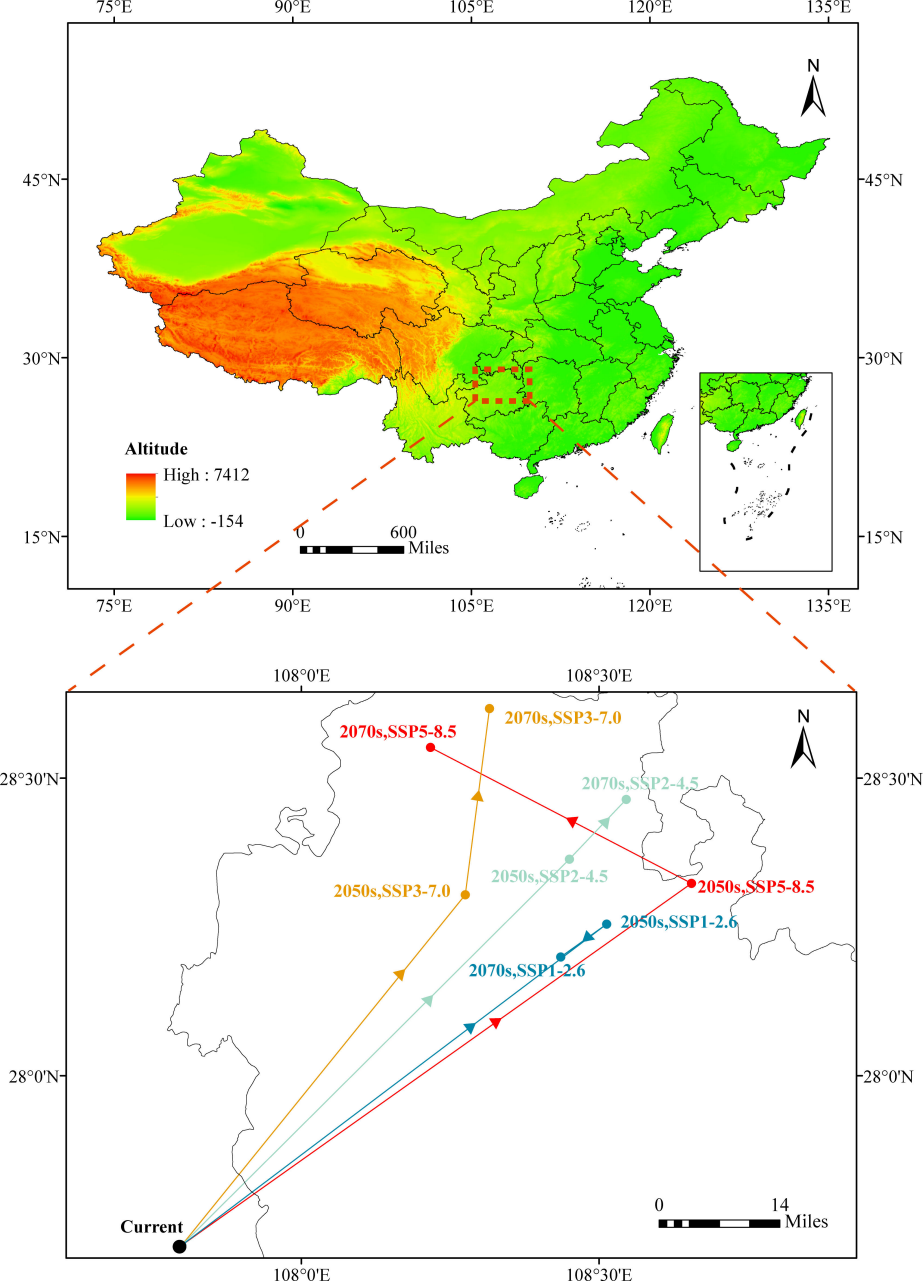
Path of the centroid of the suitable habitat distribution center under future climate scenarios.

The current suitability center of *C*Las is marked by a black dot and situated in Fenggang County, Zunyi City, northeastern Guizhou Province (107.90E, 27.71N). The projected climate change indicates a northeastward migration of the suitability center within Guizhou Province to Tongren City. Under the SSP1-2.6, SSP2-4.5, SSP3-7.0, and SSP5-8.5 scenarios, the suitability centers for *C*Las in the 2050s are projected to be in Yinjiang County (108.51E, 28.25N), Yanhe County (108.45E, 28.36N), Dejiang County (108.28E, 28.30N), and Songtao County (108.66E, 28.32N), respectively. Moreover, the suitability centers for *C*Las in 2070 are expected to be in Yinjiang County (108.44E, 28.20N), Yanhe County (108.54E, 28.47N), and Yanhe County (108.32E, 28.62N). Inevitably, it is observed that the center of suitable living areas for *C*Las will shift toward higher latitudes and longitudes in the future (Fig. 7).

## DISCUSSION

Phages are viruses that attack bacteria and incorporate their genetic material, conferring biological traits on the host bacterium (36). This integration involves inserting their genetic material into the host’s chromosomes, enabling them to utilize the host cell’s biosynthetic machinery for replication. The phage genome is subsequently transmitted to successive bacterial generations during the host’s replication process. The integration and replication of prophage genomes are interdependent on the host bacterial genome, and these integrated genes often play a role in the infectivity and pathogenicity of bacteria (14, 16).

Genetic diversity plays a crucial role in the survival and adaptation of species to environmental challenges, such as cold, drought, and high altitudes (21, 22). Different environmental factors selectively favor genotypes better suited for survival in specific conditions. Over extended periods, natural selection processes can result in significant variations in population genetic diversity. Studying population genetic diversity aids in understanding various aspects of biological evolution, including species formation, genetic drift, gene flow, and others. This knowledge contributes to our understanding of biodiversity and ecosystems and facilitates the conservation and management of species resources (25, 27, 37, 38).

Previous studies employing prophage typing have revealed the predominance of *C*Las strains carrying a single prophage among populations collected in southern China from 2010 to 2019. Specifically, the type 2 prophage carried by *C*Las accounted for approximately 67.0% of the total samples. In populations with multiple prophages, strains carrying a combination of type 1 and type 3 prophages were most prevalent, constituting 14.4% of the samples (23). The study yielded comparable findings, with a predominant presence of single prophage-carrying *C*Las strains, particularly those with the type 2 prophage, constituting 55% of all samples. Among strains with multiple prophages, those carrying a combination of type 1 and type 2 prophages were the most common (9.2%), followed by strains with a combination of type 1 and 3 prophages (8.4%). According to Fu et al. (28), *C*Las populations in mainland China can be categorized into two geographic regions based on altitude: low-altitude regions (LAR) and high-altitude regions (HAR). LAR primarily includes the Guangdong, Fujian, Guangxi, Jiangxi, Zhejiang, and Hunan provinces. Nonetheless, HAR encompasses the Yunnan, Sichuan, and Guizhou provinces. Their study found that the genetic diversity of the *C*Las populations in Sichuan province from HAR and the Guangdong, Fujian, and Guangxi provinces from LAR was relatively low. Meanwhile, the genetic diversity of the *C*Las populations in Yunnan province from HAR and Jiangxi, Zhejiang, and Hunan provinces from LAR was higher (28).

Our investigation uncovered heterogeneity in the genetic diversity among *C*Las populations at various loci. We speculate that the genetic structure of the species exhibits correlation with diverse environments, leading to varying evolutionary rates across distinct loci. The sequence analysis of the identified prophages revealed an elevated haplotype diversity in *C*Las populations in Yunnan and Sichuan provinces, implying the existence of unique lineages. This finding was further supported by the network graph of the SC1-gp030 haplotype, conceivably attributed to their close geographical locations, similar altitudes, climatic factors, or potential different origins (39). For instance, the distinctive geographic and climatic features of the Qinghai–Tibet Plateau have played a significant role in driving species formation and genetic differentiation in populations (40). Moreover, a study identified a residual prophage, named *C*LasMV1, through metagenomic analysis, and although the complete sequence was not obtained, it has been confirmed to be widely present in the *C*Las population in Guangdong province. In this study, type 1, 2, and 3 prophages were not detected in 51 *C*Las samples, indicating the possible presence of this *C*LasMV1 prophage or other types of novel prophages in these samples (41).

*C*Las has a history spanning over 100 years in China, during which its genetic diversity likely evolved. Previous analyses using chromosomal gene markers indicated a relatively low genetic diversity in *C*Las, which has been attributed to the conservative nature of its chromosomal genes (37). Despite evidence of a population expansion in China (42, 43), no clear systematic geographical structure has been identified. These findings both align with and differ from the results of our study. Our research revealed that all three types of prophages exhibited a reduced genetic diversity, with type 1 prophages showing a relatively higher diversity compared to type 2 and 3 prophages. Neutral tests and mismatch distribution curves suggested that type 2 and 3 prophages carried by *C*Las underwent population expansion, whereas type 1 prophages remained relatively stable. This stability may be attributed to the northward migration of *D. citri* carrying *C*Las with the type 2 and 3 prophages in recent years. Climate change has expanded the distribution of *D. citri*, accelerated their development, increased their flight performance, and enhanced their dispersal rate, leading to complex diffusion and damage patterns of *C*Las on both temporal and spatial scales (20, 44, 45). *C*Las primarily parasitizes the phloem of *Rutaceae* plants. Therefore, the recent expansion of citrus cultivation areas has increased the potential survival ability of *C*Las (46). The incidence of *C*Las is influenced by both seasonal factors and altitude (21–24). In this study, bio4 (temperature seasonality) and bio20 (altitude) emerged as the two most influential variables in MaxEnt, aligning with the observed prevalence pattern of *C*Las. Previous studies utilizing MaxEnt to predict the global climate suitability of *C*Las, primarily focusing on the United States, emphasized bio12 (annual precipitation) and bio6 (min temperature of the coldest month) as the two most contributing variables (47). This finding contrasts with our results, suggesting a potential discrepancy arising from the omission of altitude variables in prior studies. Nevertheless, earlier findings consistently indicate that southern China is highly conducive to the establishment of *C*Las, supporting and reinforcing our present results (47). With the impact of future global warming, the suitability range of *C*Las in China is expected to increase, and the center of the *C*Las habitat suitability will shift toward the northeast. Currently, some citrus-growing areas have not yet detected *C*Las; however, they carry a high to moderate risk of *C*Las invasion in the future, such as Chongqing Municipality and Hubei Province. Therefore, future prevention and control efforts should prioritize these regions.

In summary, this study conducted sequencing of three distinct types of prophage genes, revealing the genetic variability among different prophages in China. Furthermore, this research also illustrated the potential impact of climate change on the transmission risk of *C*Las. Significantly, it provides a framework for understanding the distribution of *C*Las in China, thereby facilitating the implementation of proactive management interventions. The findings of this study have the potential to contribute to epidemiological investigations and the development of efficient strategies for controlling HLB.

## MATERIALS AND METHODS

### Sample collection

**Fig 8:**
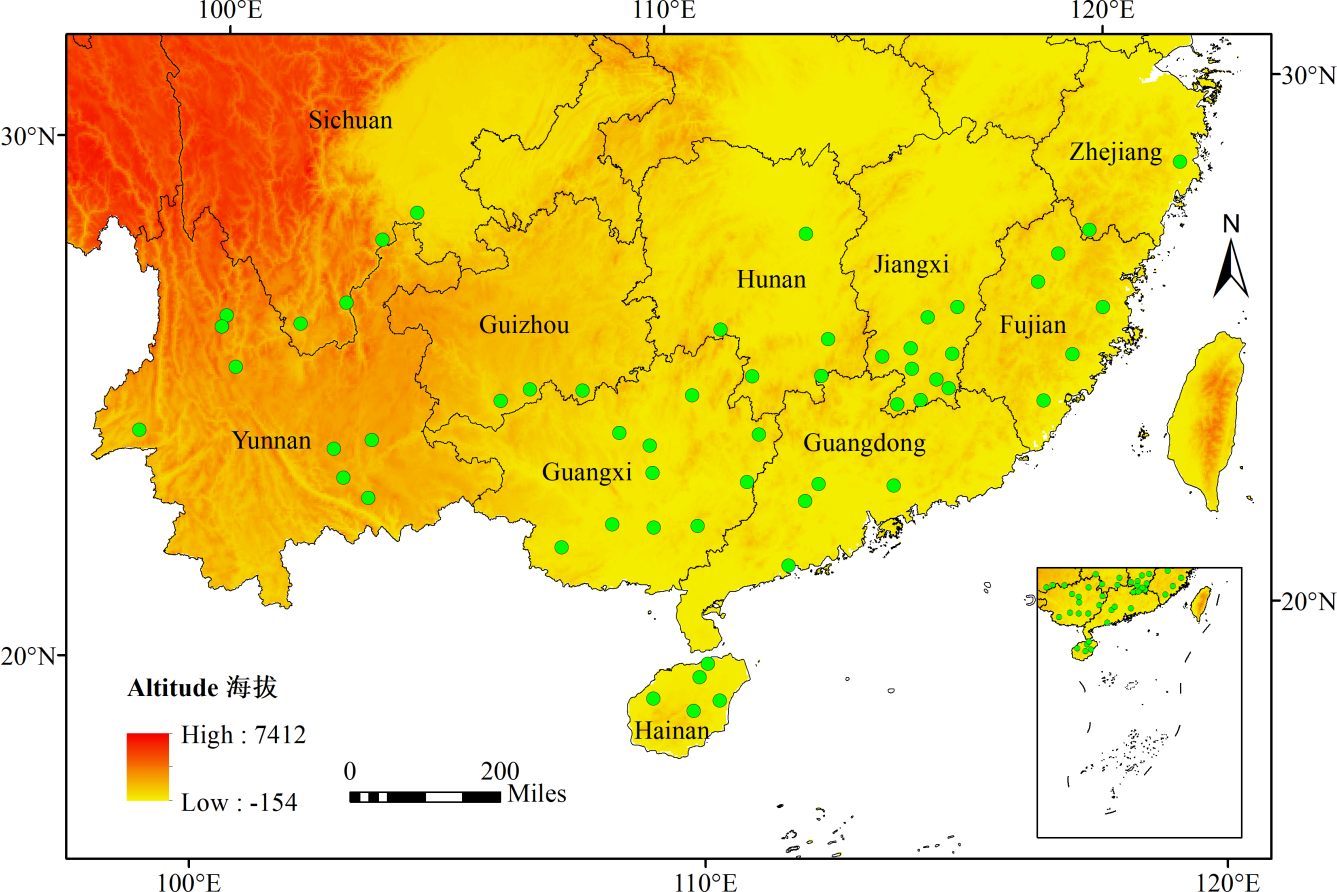
Geographical locations indicated by green dots represent the provinces from which the *C*Las samples were collected.

From 2017 to 2023, a survey was conducted in 10 provinces (Hainan (HN), Yunnan (YN), Sichuan (SC), Guangxi (GX), Guangdong (GD), Fujian (FJ), Zhejiang (ZJ), Guizhou (GZ), Hunan (HN), and Jiangxi (JX)) of China to collect citrus leaves infected with *C*Las. The citrus leaves were collected by visually identifying symptoms of HLB, such as mottled yellowing of the leaves. Genomic DNA extraction from the collected citrus leaves was performed using the cetyltrimethylammonium bromide method. For the HLB infection confirmation, OI1/OI2cprimers were employed in the PCR for identification purposes (48). A total of 500 HLB-positive individuals from the 10 provinces were selected for prophage type identification and genetic diversity analysis. The sample collection sites are visually represented in Fig. 8 and further detailed in Table S4.

### Identification for prophages of *C*Las

To identify the prophage types of *C*Las, the specific primers listed in Table S5 were used in a PCR reaction containing 1 µL genomic DNA, 0.5 µL each of forward and reverse primers, 10 µL 2 × Taq PCR mix (Tiangen Biotech Co., Ltd., Beijing, China), and 8 µL ddH_2_O. The thermal cycling conditions consisted of an initial denaturation step at 94°C for 4 min, followed by 35 cycles of denaturation at 94℃ for 30 s, annealing at the primer-specific temperature for 30 s (refer to Table S5), extension at 72°C for 90 s, and a final extension step at 72°C for 10 min (16). Following PCR amplification, the products were separated on a 1.5% agarose gel and visualized using a Bio-Rad gel documentation system (Bio-Rad Laboratories, Inc., Hercules, CA, USA). The amplified products were then purified via the Tiangen Purification Kit (Tiangen Biotech Co., Ltd., Beijing, China). Bidirectional sequencing of all amplified products was conducted using the Sanger method at Sangon Biotech Co., Ltd. (Shanghai, China), with the same primers used for both amplification and purification.

### Haploid network and phylogenetic analysis

We obtained sequences using Bioedit 7.2.5 (49) and aligned the sequences of SC1-gp035, SC2-gp030, and P-JXGC-gp08 using MEGA X (50). DnaSP 5.0 (51) software was employed to calculate nucleotide diversity, haplotype diversity, and fixed index of genetic differentiation (*F*_st_) between populations for each gene sequence. Neutral tests and mismatch distributions were conducted using DnaSP 5.0 (51) software, with Tajima’s *D*, Fu and Li’s *D*, and Fu and Li’s *F* values calculated to infer population expansion and contraction trends. To analyze the genetic evolutionary relationship among different geographical populations of *C*Las, we constructed a haplotype network branch diagram using PopART 1.7 software (52). We downloaded seven prophage sequences of *C*Las from NCBI (https://www.ncbi.nlm.nih.gov/, accessed on 12 August 2023,). These sequences, with accession numbers HQ377372.1, NC019549.1, HQ377374.1, MN660061.1, KY661963.1, NC019550.1, and HQ3773774.1, were combined with representative haplotypes obtained in our study for phylogenetic analysis. The phylogenetic tree was constructed using the H + K + Y model with 1000 bootstrap replicates (50).

### Habitat suitability analysis of *C*Las

This study collected distribution records of 56 *C*Las and obtained 349 occurrence and distribution records from the official website of the Ministry of Agriculture and Rural Affairs of the People’s Republic of China (http://www.moa.gov.cn/govpublic/ZZYGLS/202309/t20230904_6435699.htm, accessed on 12 September 2023). The distribution records obtained were georeferenced using Baidu Maps (https://api.map.baidu.com/lbsapi/getpoint/index.html, accessed on 10 September 2023). To address issues related to spatial autocorrelation and pseudo-random replication in species occurrence data, ENMTools 1.4 software (53) was utilized. This ensured that each 5 km^2^ grid cell contained only one distribution record. Consequently, a comprehensive data set consisting of 386 records was obtained (Fig. S1), representing the occurrence of *C*Las species.

Twenty environmental variables were collected, including 19 bioclimatic variables (bio 1–bio 19) and one terrain variable (bio 20), and chosen as candidate variables to be used for modeling (Table S6). Climate data for the periods 1970–2000 (near current), 2041–2060 (2050s), and 2060–2080 (2070s) were obtained from WorldClim version 2.1 with a resolution of 2.5 arc minutes (https://www.worldclim.org/data/index.html, accessed on 11 September 2023). The BCC-CSM2-MR global climate model data were used for projecting the potential distribution of *C*Las in the 2050s and 2070s under four shared socioeconomic pathways (subsp.) in CMIP6 (phase 6 of the Coupled Model Intercomparison Project). These subsp. include SSP1-2.6, SSP2-4.5, SSP3-7.0, and SSP5-8.5, each representing different levels of greenhouse gas emissions.

To mitigate the multicollinearity between the selected variables, we initially entered 20 environmental variables into MaxEnt for preliminary modeling. We utilized 25% of the data as the testing subset for bootstrap sampling, repeating the process 10 times for model inference. Subsequently, we ranked the contribution rates of each environmental variable to the model results. Using ENMTools 1.4 software (53), we calculated the Pearson correlation coefficient (r) for environmental variables (Fig. S2). For highly correlated variables (*r* ≥ | 0.8 |), we retained only those that significantly contributed to the model results. Accordingly, we identified seven variables (Bio2, Bio3, Bio4, Bio5, Bio17, Bio15, and Bio20) for establishing the model.

Feature combination (FC) and regularization multiplier (RM) are the two most crucial model parameters in the MaxEnt model (34). Optimizing the selection of these two parameters can significantly enhance the model’s prediction accuracy. The FC consists of five features: linear (L), quadratic (Q), product (*P*), threshold (T), and hinge (H). To optimize the MaxEnt model, the RM values range from 0.1 to 6, increasing by 0.5 per run, starting from 0.5. The feature combination is set to 6, namely, L, LQ, H, LQH, LQHP, and LQHPT. The ENMeval package in the R software on 4.1.2 version is used to test all the mentioned combinations (54). The optimal feature combination is identified as LQHPT, and the optimal normalization factor is determined to be 1.5 (Fig. S3).

Finally, 75% of the distribution points were used as the training set, and the remaining 25% were allocated as the test set. Ten repetitions were performed on the model, and the average of these repeats was calculated to predict a potentially suitable area. The receiver operating characteristic (ROC) curve was employed to evaluate the model’s performance, with the area under the curve (AUC) threshold set at 0.96, indicating the overall satisfactory performance of the simulation results (Fig. S4). Utilizing the predicted probability of occurrence, we categorized potentially suitable areas into four tiers: unsuitable habitat (<0.01), less suitable habitat (0.01–0.1), moderately suitable habitat (0.1–0.2), and highly suitable habitat (>0.2) (55).

## SUPPLEMENTAL MATERIAL

Supplemental figures and tables spectrum.00633-24-s0001.docx Fig. S1 to S4; Tables S1 to S6.

ASM does not own the copyrights to Supplemental Material that may be linked to, or accessed through, an article. The authors have granted ASM a non-exclusive, world-wide license to publish the Supplemental Material files. Please contact the corresponding author directly for reuse.
