# Supplementary material for: Potential habitat suitability of Candidatus Liberibacter asiaticus and genetic diversity of its prophages across China
Source: Microbiol Spectr. 2024 Sep 24;12(11):e00633-24. doi: 10.1128/spectrum.00633-24 (PMC11537051; doi:10.1128/spectrum.00633-24)
Supplement: Supplemental figures and tables — Fig. S1 to S4; Tables S1 to S6. [file spectrum.00633-24-s0001.docx]

Table S1 The distribution of different types of proophage combinations in *C*Las samples collected in China.

| **Prophage types** | **Guangdong** | **Guangxi** | **Sichuan** | **Guizhou** | **Yunnan** | **Hainan** | **Jiangxi** | **Fujian** | **Zhejiang** | **Hunan** | **Total** |
| --- | --- | --- | --- | --- | --- | --- | --- | --- | --- | --- | --- |
| Type1 | 2(4%) | 1(2%) | 7(14%) | 2(4%) | 21(42%) | 4(8%) | 1(2%) | 0 | 5(10%) | 1(2%) | 44(8.8%) |
| Type2 | 35(70%) | 35(70%) | 2(4%) | 12(24%) | 9(18%) | 24(48%) | 40(80%) | 36(72%) | 36(72%) | 46(92%) | 275(55%) |
| Type3 | 2(4%) | 2(4%) | 1(2%) | 0 | 0 | 1(2%) | 0 | 7(14%) | 0 | 0 | 13(2.6%) |
| Type1 + Type2 | 9(18%) | 0 | 3(6%) | 19(38%) | 6(12%) | 4(8%) | 2(4%) | 0 | 1(2%) | 2(4%) | 46(9.2%) |
| Type1 + Type3 | 0 | 3(6%) | 24(48%) | 4(6%) | 4(8%) | 5(10%) | 1(2%) | 1(2%) | 0 | 0 | 42(8.4%) |
| Type2 + Type3 | 0 | 3(6%) | 0 | 0 | 0 | 0 | 0 | 2(4%) | 0 | 0 | 5(1%) |
| Type1 + Type2 + Type3 | 0 | 0 | 8(16%) | 13(26%) | 0 | 3(6%) | 0 | 0 | 0 | 0 | 24(4.8%) |
| NONE | 2(4%) | 6(12%) | 5(10%) | 0 | 10(20%) | 9(18%) | 6(12%) | 4(8%) | 8(16%) | 1(2%) | 51(10.2%) |

Table S2 diversity of different prophage loci of *C*Las in different provinces..

| Locus | Province | N | S | H | Hd | Pi | K |
| --- | --- | --- | --- | --- | --- | --- | --- |
| SC1_gp035 | Guangdong | 11 | 11 | 7 | 0.89091 | 0.00262 | 2.36364 |
|  | Guangxi | 4 | 0 | 1 | 0 | 0 | 0 |
|  | Hainan | 16 | 1 | 2 | 0.12500 | 0.00014 | 0.12500 |
|  | Sichuan | 42 | 27 | 7 | 0.41812 | 0.00198 | 1.78513 |
|  | Guizhou | 38 | 0 | 1 | 0 | 0 | 0 |
|  | Yunnan | 31 | 26 | 3 | 0.12688 | 0.00319 | 2.88172 |
|  | Fujian | 2 | 0 | 1 | 0 | 0 | 0 |
|  | Zhejiang | 6 | 0 | 1 | 0 | 0 | 0 |
|  | Jiangxi | 4 | 0 | 1 | 0 | 0 | 0 |
|  | Hunan | 3 | 0 | 1 | 0 | 0 | 0 |
| SC2_gp040 | Guangdong | 44 | 0 | 1 | 0 | 0 | 0 |
|  | Guangxi | 38 | 0 | 1 | 0 | 0 | 0 |
|  | Hainan | 48 | 0 | 1 | 0 | 0 | 0 |
|  | Sichuan | 13 | 4 | 5 | 0.53846 | 0.00084 | 0.61538 |
|  | Guizhou | 44 | 1 | 2 | 0.04545 | 0.00006 | 0.04545 |
|  | Yunnan | 15 | 2 | 3 | 036190 | 0.00052 | 0.38095 |
|  | Fujian | 38 | 0 | 1 | 0 | 0 | 0 |
|  | Zhejiang | 37 | 0 | 1 | 0 | 0 | 0 |
|  | Jiangxi | 42 | 0 | 1 | 0 | 0 | 0 |
|  | Hunan | 48 | 0 | 1 | 0 | 0 | 0 |
| P_JXGC_gp08 | Guangdong | 2 | 0 | 1 | 0 | 0 |  |
|  | Guangxi | 8 | 5 | 3 | 0.46429 | 0.00145 | 0.12500 |
|  | Hainan | 9 | 2 | 3 | 0.55556 | 0.00071 | 0.61111 |
|  | Sichuan | 33 | 1 | 2 | 0.06061 | 0．00007 | 0.06061 |
|  | Guizhou | 17 | 0 | 1 | 0 | 0 | 0 |
|  | Yunnan | 4 | 0 | 1 | 0 | 0 | 0 |
|  | Fujian | 10 | 0 | 1 | 1 | 0 | 0 |
|  | Jiangxi | 8 | 5 | 3 | 0.46429 | 0.00145 | 0.12500 |

Table S3 The contribution rates of selected environment variables affecting the distribution of *C*Las.

| **Variable** | **Percent contribution(%)** | **Permutation importance(%)** |
| --- | --- | --- |
| bio4 | 37.9 | 45.7 |
| bio17 | 36.3 | 4.5 |
| bio5 | 11.7 | 4.2 |
| bio20 | 10.2 | 40.4 |
| bio3 | 1.7 | 3.8 |
| bio2 | 1.7 | 0.6 |
| bio15 | 0.5 | 0.8 |

TableS4 Geographical location and numbers of “Candidatus Liberibacter asiaticus” (*C*Las) samples collected from each province in this study

| **Province** | **Location** | | | **Longitude** | **Latitude** | **Numbers** |
| --- | --- | --- | --- | --- | --- | --- |
| Fujian province | Nanping city | | | 118.3271 | 27.27537 | 3 |
|  | Yongchun city | | | 118.3413 | 25.31988 | 25 |
|  | Minhou city | | | 119.1253 | 26.13554 | 8 |
|  | Zhangzhou city | | | 117.6066 | 24.50578 | 1 |
|  | Shunchang county | | | 117.8119 | 26.78866 | 13 |
| Hainan province | Chengmai county | | | 110.0566 | 19.72502 | 11 |
|  | Qionghai city | | | 110.4385 | 19.24378 | 9 |
|  | Haikou city | | | 110.2387 | 19.97844 | 11 |
|  | Qiongzhong county | | | 109.9072 | 19.07005 | 13 |
|  | Changjiang county | | | 109.1182 | 19.34794 | 6 |
| Yunan province | Lijiang city | | | 100.2310 | 26.77489 | 12 |
|  | Jianshui county | | | 102.8280 | 23.72501 | 14 |
|  | Dehong city | | | 98.50569 | 24.47775 | 3 |
|  | Heqing county | | | 100.1382 | 26.55353 | 2 |
|  | Bingchuan county | | | 100.4738 | 25.79232 | 8 |
|  | Mile city | | | 103.4077 | 24.46457 | 2 |
|  | Honghe city | | | 103.3579 | 23.33974 | 7 |
|  | Yuxi city | | | 102.6133 | 24.27947 | 2 |
| Hunan province | Daoxian county | | | 111.5028 | 25.50775 | 2 |
|  | Yizhang county | | | 112.9766 | 25.42203 | 17 |
|  | Changsha city | | | 112.8865 | 28.1617 | 1 |
|  | Xinning county | | | 110.8922 | 26.44309 | 13 |
|  | Yongxing county | | | 113.1875 | 26.1187 | 17 |
| Sichuan province | Yibing city | | | 104.3383 | 28.83655 | 6 |
|  | Ningnan county | | | 102.8110 | 27.09685 | 5 |
|  | Panzhihua city | | | 101.8310 | 26.66733 | 33 |
|  | Leibo county | | | 103.5738 | 28.31196 | 6 |
| Guangxi province | Liuzhou city | | | 109.2597 | 24.28059 | 5 |
|  | Hechi city | | | 108.6333 | 24.55089 | 3 |
|  | Laibing city | | | 109.2941 | 23.75575 | 7 |
|  | Nanning city | | | 108.4148 | 22.78236 | 6 |
|  | Yulin city | | | 110.1793 | 22.68144 | 6 |
|  | Guilin City | | | 110.2046 | 25.21634 | 7 |
|  | Heng County | | | 109.2681 | 22.68957 | 5 |
|  | Wuzhou City | | | 111.2527 | 23.47983 | 4 |
|  | Hezhou City | | | 111.5655 | 24.38339 | 6 |
|  | Chongzuo City | | | 107.3571 | 22.36520 | 1 |
| Guangdong province | Zhaoqing City | | | 112.4364 | 23.03613 | 11 |
|  | Yangjiang City | | | 111.9971 | 21.80449 | 11 |
|  | Boluo county | | | 114.2914 | 23.19023 | 20 |
|  | Sihui City | | 112.7389 | | 23.33955 | 8 |
| Jiangxi province | Xinfeng county | 114.9111 | | | 25.39955 | 5 |
|  | Xunwu county | 115.6495 | | | 24.95818 | 2 |
|  | Quannan county | 114.5291 | | | 24.74322 | 4 |
|  | Chongyi county | 114.3036 | | | 25.69264 | 12 |
|  | Ganzhou city | 114.9273 | | | 25.79589 | 13 |
|  | Dingnan county | 115.0265 | | | 24.78691 | 2 |
|  | Ningdu county | 116.023 | | | 26.48917 | 3 |
|  | Huichang county | 115.8002 | | | 25.60474 | 3 |
|  | Xingguo county | 115.3621 | | | 26.35139 | 2 |
|  | Anyuan county | 115.4040 | | | 25.15138 | 4 |
| Zhejiang province | Taizhou city | 121.2697 | | | 28.66066 | 13 |
|  | Qingyuan county | 119.077 | | | 27.64105 | 37 |

Table S5 The information of gene regions and primer pairs used in the current study.

| **Prophage types** | **Locus** | **Primers** | **Sequence (5′–3′)** | **Tm(℃)** |
| --- | --- | --- | --- | --- |
| Type1 | SC1_gp035 | F | TGGCTCGGGTTCAGGTAAAT | 60 |
|  |  | R | AAGGGCGACGCATGTATTTC |  |
| Type2 | SC2_gp030 | F | ACCCTCGCACCATCATGTTA | 60 |
|  |  | R | TCGTCTTGATTGGGCAGAGT |  |
| Type3 | P_JXGC_gp08 | F | CTGATCCTTTACCATGCCGC | 60 |
|  |  | R | CAGCGAAACCGATCTTGAGG |  |

Table S6 The information of gene regions and primer pairs used in the current study.

| **Type** | **Code** | **Variable name** |
| --- | --- | --- |
| Climatic | bio1 | Annual mean temperature |
|  | bio2 | Mean diurnal range (monthly mean (max temp minus min temp)) |
|  | bio3 | Isothermality (bio2/bio7) (×100) |
|  | bio4 | Temperature seasonality (standard deviation ×100) |
|  | bio5 | Max temperature of warmest month |
|  | bio6 | Min temperature of coldest month |
|  | bio7 | Temperature annual range (bio5 minus bio6) |
|  | bio8 | Mean temperature of wettest quarter |
|  | bio9 | Mean temperature of driest quarter |
|  | bio10 | Mean temperature of warmest quarter |
|  | bio11 | Mean temperature of coldest quarter |
|  | bio12 | Annual precipitation |
|  | bio13 | Precipitation of wettest month |
|  | bio14 | Precipitation of driest month |
|  | bio15 | Precipitation seasonality (coefficient of variation) |
|  | bio16 | Precipitation of wettest quarter |
|  | bio17 | Precipitation of driest quarter |
|  | bio18 | Precipitation of warmest quarter |
|  | bio19 | Precipitation of coldest quarter |
| Topographical | bio20 | Elevation |


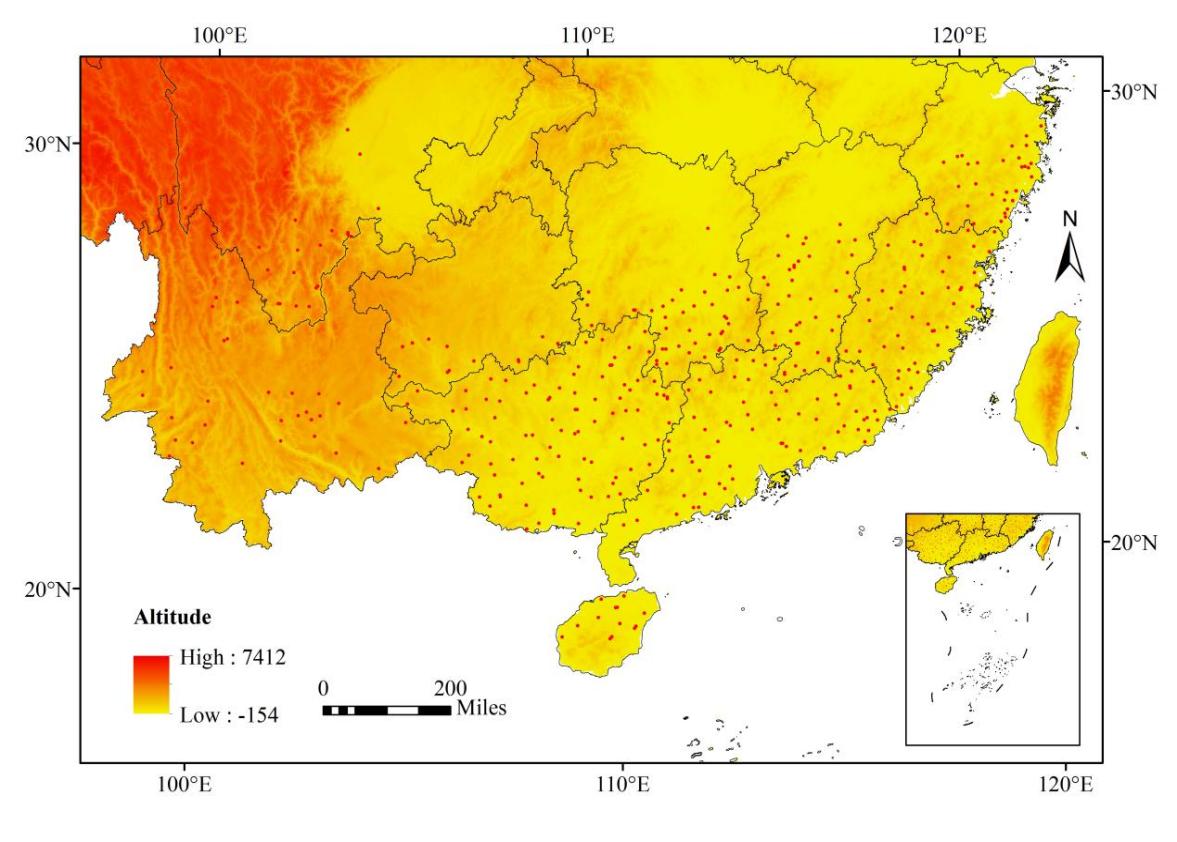


Figure S1 386 *C*Las distribution points obtained after screening were used for MaxEnt analysis


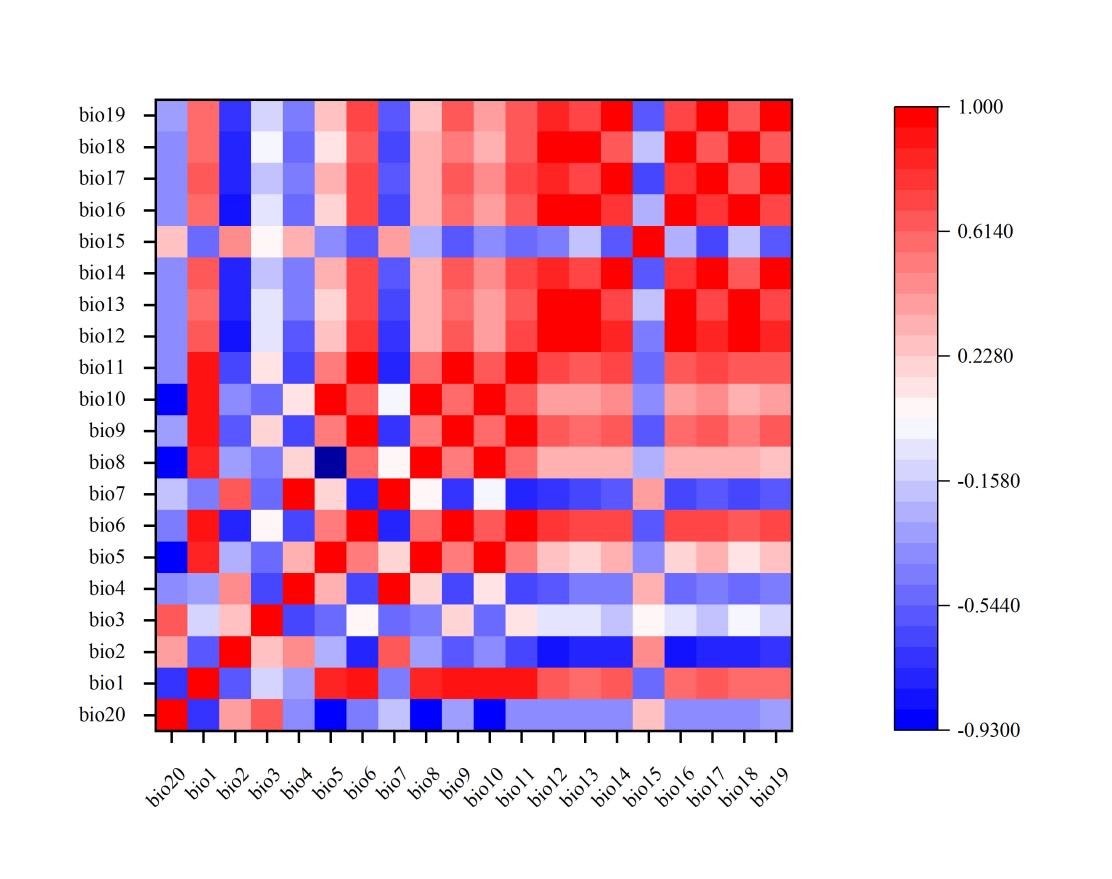


Figure S2 Environmental variable correlation heatmap


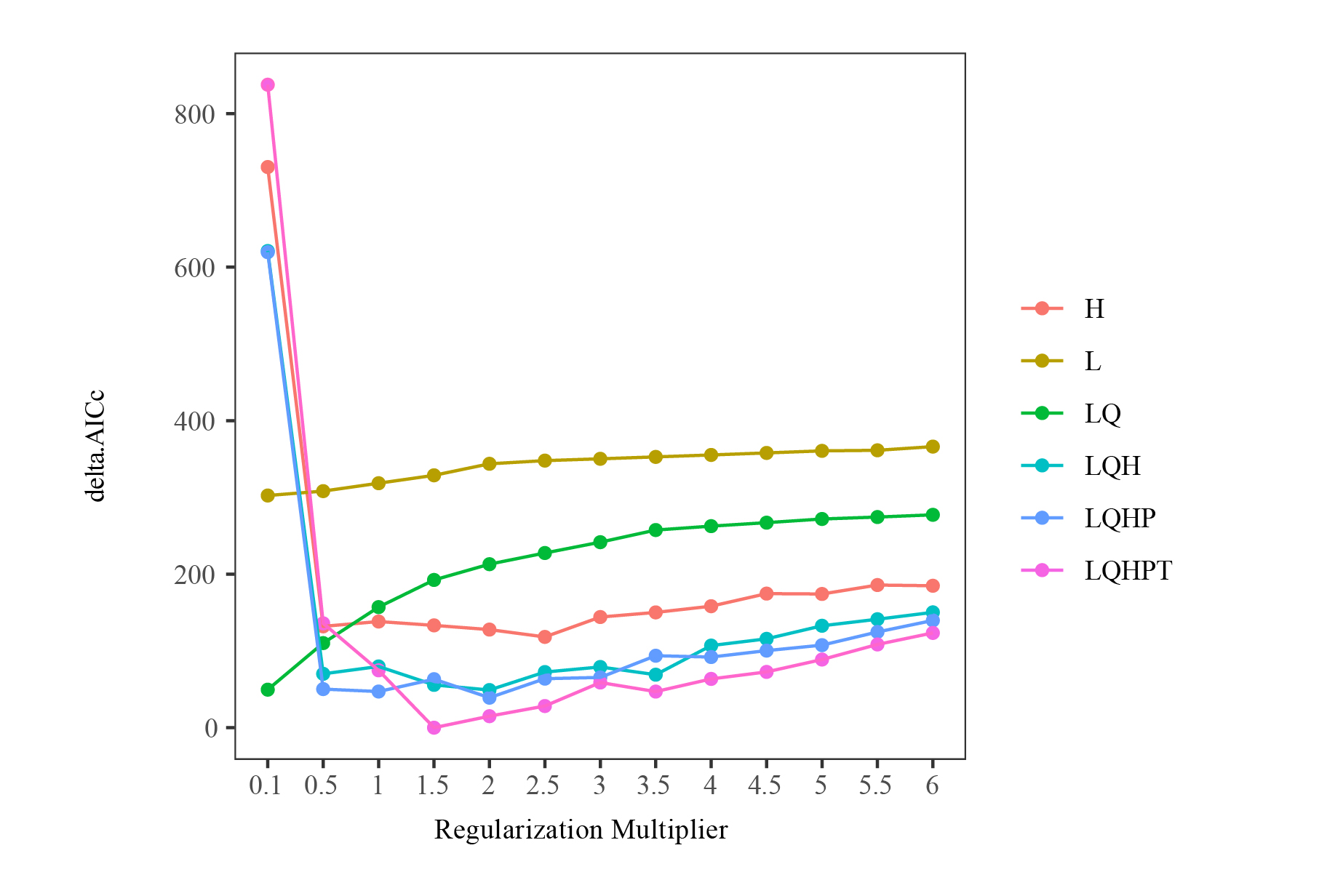


Figure S3 Optimization results of MAXENT model parameters


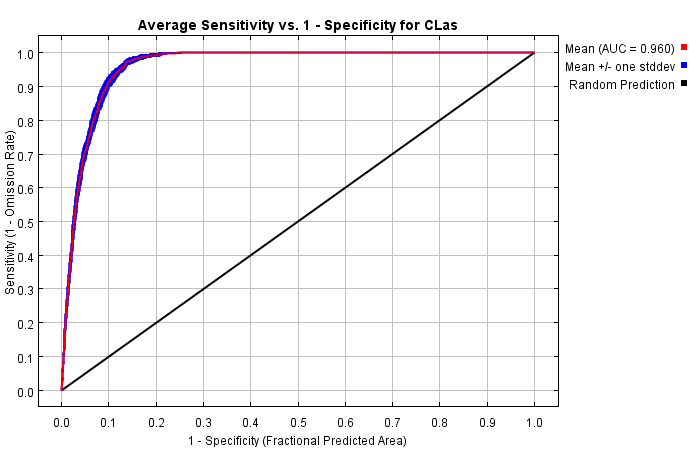


Figure S4 Receiver operating characteristic (ROC) curve of the MaxEnt niche model

| Province  省份 | Location | | Longitude | Latitude | Numbers |
| --- | --- | --- | --- | --- | --- |
| Fujian province | Nanping city | | 118.3271 | 27.27537 | 3 |
|  | Yongchun city | | 118.3413 | 25.31988 | 25 |
|  | Minhou city | | 119.1253 | 26.13554 | 8 |
|  | Zhangzhou city | | 117.6066 | 24.50578 | 1 |
|  | Shunchang county | | 117.8119 | 26.78866 | 13 |
| Hainan province | Chengmai county | | 110.0566 | 19.72502 | 11 |
|  | Qionghai city | | 110.4385 | 19.24378 | 9 |
|  | Haikou city | | 110.2387 | 19.97844 | 11 |
|  | Qiongzhong county | | 109.9072 | 19.07005 | 13 |
|  | Changjiang county | | 109.1182 | 19.34794 | 6 |
| Yunan province | Lijiang city | | 100.2310 | 26.77489 | 12 |
|  | Jianshui county | | 102.8280 | 23.72501 | 14 |
|  | Dehong city | | 98.50569 | 24.47775 | 3 |
|  | Heqing county | | 100.1382 | 26.55353 | 2 |
|  | Bingchuan county | | 100.4738 | 25.79232 | 8 |
|  | Mile city | | 103.4077 | 24.46457 | 2 |
|  | Honghe city | | 103.3579 | 23.33974 | 7 |
|  | Yuxi city | | 102.6133 | 24.27947 | 2 |
| Hunan province | Daoxian county | | 111.5028 | 25.50775 | 2 |
|  | Yizhang county | | 112.9766 | 25.42203 | 17 |
|  | Changsha city | | 112.8865 | 28.1617 | 1 |
|  | Xinning county | | 110.8922 | 26.44309 | 13 |
|  | Yongxing county | | 113.1875 | 26.1187 | 17 |
| Sichuan province | Yibing city | | 104.3383 | 28.83655 | 6 |
|  | Ningnan county | | 102.8110 | 27.09685 | 5 |
|  | Panzhihua city | | 101.8310 | 26.66733 | 33 |
|  | Leibo county | | 103.5738 | 28.31196 | 6 |
| Guangxi province | Liuzhou city | | 109.2597 | 24.28059 | 5 |
|  | Hechi city | | 108.6333 | 24.55089 | 3 |
|  | Laibing city | | 109.2941 | 23.75575 | 7 |
|  | Nanning city | | 108.4148 | 22.78236 | 6 |
|  | Yulin city | | 110.1793 | 22.68144 | 6 |
|  | Guilin City | | 110.2046 | 25.21634 | 7 |
|  | Heng County | | 109.2681 | 22.68957 | 5 |
|  | Wuzhou City | | 111.2527 | 23.47983 | 4 |
|  | Hezhou City | | 111.5655 | 24.38339 | 6 |
|  | Chongzuo City | | 107.3571 | 22.36520 | 1 |
| Guangdong province | Zhaoqing City | | 112.4364 | 23.03613 | 11 |
|  | Yangjiang City | | 111.9971 | 21.80449 | 11 |
|  | Boluo county | | 114.2914 | 23.19023 | 20 |
|  | Sihui City | 112.7389 | | 23.33955 | 8 |
| Jiangxi province | Xinfeng county | 114.9111 | | 25.39955 | 5 |
|  | Xunwu county | 115.6495 | | 24.95818 | 2 |
|  | Quannan county | 114.5291 | | 24.74322 | 4 |
|  | Chongyi county | 114.3036 | | 25.69264 | 12 |
|  | Ganzhou city | 114.9273 | | 25.79589 | 13 |
|  | Dingnan county | 115.0265 | | 24.78691 | 2 |
|  | Ningdu county | 116.023 | | 26.48917 | 3 |
|  | Huichang county | 115.8002 | | 25.60474 | 3 |
|  | Xingguo county | 115.3621 | | 26.35139 | 2 |
|  | Anyuan county | 115.4040 | | 25.15138 | 4 |
| Zhejiang province | Taizhou city | 121.2697 | | 28.66066 | 13 |
|  | Qingyuan county | 119.077 | | 27.64105 | 37 |
| Guizhou province | Luodian county | 106.7603 | | 25.43632 | 30 |
|  | Wangmo county | 106.1318 | | 25.22177 | 7 |
|  | Libo county | 107.8848 | | 25.38968 | 13 |
